# Method development and validation of ten pyrethroid insecticides in edible mushrooms by Modified QuEChERS and gas chromatography-tandem mass spectrometry

**DOI:** 10.1038/s41598-020-64056-7

**Published:** 2020-04-27

**Authors:** Fajun Tian, Chengkui Qiao, Jing Luo, Linlin Guo, Tao Pang, Rongli Pang, Jun Li, Caixia Wang, Ruiping Wang, Hanzhong Xie

**Affiliations:** Zhengzhou Fruit Research Institute, Chinese Academy of Agricultural Sciences, Zhengzhou, 450009 China

**Keywords:** Mass spectrometry, Chemical safety

## Abstract

A method for simultaneous determination of ten pyrethroid insecticides residues in edible mushrooms was developed. The samples were pretreated by a quick, easy, cheap, effective, rugged (QuEChERS) method. The ten pyrethroid insecticides were extracted from six kinds of edible mushrooms using acetonitrile and subsequently cleaned up by octadecylsilane (C18) or primary secondary amine (PSA). Instrumental analysis was completed in 16 min using gas chromatography-tandem mass spectrometry (GC-MS/MS). The overall average recoveries in the six kinds of edible mushrooms at three levels (10, 100 and 1000 μg kg^−1^) ranged from 72.8% to 103.6%. The intraday and interday relative standard deviations (RSD) were lower than 13.0%. The quantification limits were below 5.57 μg kg^−1^ in different matrices. The results demonstrated that the method is convenient for the quick detection of pyrethroid insecticides in edible mushrooms.

## Introduction

Edible mushrooms are considered as a delicacy with high nutritive value and unique flavor, and they are also recognized as nutraceutical foods^1,2^. More importantly than all of that, it is accepted as healthy food with a good deal of medicinal functions and important positive health function^3^. They are more and more popular with consumers and have been regarded as ingredient of gourmet cuisine all over the world. *Pleurotus ostreatus* (oyster mushroom), *Lentinus edodes* (shiitake mushroom), *Pleurotus eryngii* (eryngii mushroom), *Agaricus bisporus* (crimini mushroom), *Flammulina velutiper* (enoki mushroom), and *Hypsizygus marmoreus* (bunashimeji mushroom) are the six of the most cultivated edible mushrooms. They are quite rich in protein, essential amino acids, fiber, chitin, vitamins and other substances^4–7^. These ingredients increase the value of these edible mushrooms. In China, edible mushroom production reached 7,868,782 ton in 2017. And the mushroom production accounted for 76.8% of the world^2^. And it is the most important producers and exporters of mushrooms. However, in recent years, the pests and diseases has become very serious with the expansion of cultivation scale of six edible mushrooms^8^. Sciarid flies and Cecid are the most important pests in mushroom throughout the world^9^. It was reported that these pests have caused significant economic losses in the mushroom industry^8^. Therefore, many insecticides were used on mushrooms to control these pests which can boost yields and reduce the economic losses. So, detailed investigations on pesticide residues in mushrooms are very important to reduce the use of pesticides and ensure food safety.

Many types of synthetic insecticides are frequently used to control pests in mushroom cultivation throughout the world^10–12^. Because pyrethroids has high level of effectiveness, broad spectrum of effects and low toxicity, it has been widely used in the production all over the world^13,14^. In 2015, pyrethroids was among the most important classes of insecticides in crop, and it accounted for 38% of the world insecticide market^12^. Therefore, it is not surprised that pyrethroid residues are frequently detected in different vegetables, fruits, soil and other matrices worldwide^15^. For example, Ding *et al*. reported that the highest concentration of pyrethroid residues in vegetables in Zhejiang province reached 330 μg kg^−1^^16^. To prevent and control Sciarid and Cecid in the process of mushroom cultivation, pyrethroids are frequently used on mushrooms to boost yields. Therefore, determination of pyrethroid residues in mushrooms is important for food safety and normal foreign trade.

As far as we know, many analytical methods have been reported for the determination of pyrethroids in various matrices. Due to co-extraction of highly complex components, such as protein, sterols, essential amino acids, and polysaccharide, extracting pyrethroids residues from edible mushrooms is difficult^2^. In the extract, these components seriously interfere with the determination of the pyrethroids. Therefore, pretreatment technology is very important in the detection of pyrethroids residues in edible mushrooms^17^. Usually, a solid-liquid extraction followed by purification is a preliminary sample preparation for the determination of pesticide residues in edible mushrooms. The purification techniques, including solid-phase extraction (SPE), gel permeation chromatography (GPC), and matrix solid-phase dispersion, are the most commonly used techniques for pretreatment procedures^18–20^. However, these sample preparation methods are complicated and use larger amounts of organic solvents^21,22^. In recent years, the QuEChERS methodology has been develpoed as a very popular method to determine pesticide residues in all kinds of food matrices^23–29^. And there are some papers that applied QuEChERS for the extraction of pesticides from mushrooms and determination of residue used GC, GC-MS/MS^30,31^, or LC-MS/MS^10,11^. However, simultaneous determination of pyrethroid residues in mushrooms by GC-MS/MS has not been reported. Moreover, GC-MS/MS is becoming more and more popular in routine pesticide residue analysis because of fewer co-matrix effects resulting in sensitive identification and the reagents costing less compared with HPLC/UPLC-MS/MS^32^.

Therefore, the aim of this paper is focused on the investigation of a rapid and effective extraction procedure using a modified QuEChERS method for simultaneously analyzing pyrethroids in various edible mushrooms (oyster mushroom, shiitake mushroom, eryngii mushroom, crimini mushroom, enoki mushroom and bunashimeji mushroom). Various extraction solvents and cleanup sorbents were studied for optimizing the pretreatment method to obtain higher recoveries. The developed method was successfully used to analyze authentic samples.

## Results and Discussion

### Optimization of GC-MS/MS parameters

The pyrethroid insecticides involved were monitored in the full scanning mode in the range *m/z* 50–600 to describe its scanning mass spectrogram and retention time. Then, the multiple reaction monitoring (MRM) transitions were optimized. The purpose of selecting precursor ions was to achieve a compromise between selectivity (the highest *m/z* ion is preferred) and sensitivity (the highest abundance ion). The method was divided into as many time segments as possible to achieve the maximum signal for each compound. The MS/MS transition was selected according the highest response for the each target compound. The run time of these pesticides were completed in 16 min. All the parameters for precursor ions, product ion, corresponding collision energies, and other optimal conditions were determined and shown in Table 1.

**Table 1:** Details of the MS/MS parameters for analysis of the compounds.

Compounds	Molecular formula	Molecular weight	Precursor ion (m/z)	Product ion (m/z)	Q/q ^a^	Collision energy (v)	Dwell time (s)	Retention time (min)
Bifenthrin	C_23_H_22_ClF_3_O_2_	422.9	181.2	166.2	Q	10	24	11.54
			181.2	165.2	q	20	24	
Fenpropathrin	C_22_H_23_NO_3_	349.4	207.9	181.0	Q	5	24	11.63
			264.9	210.0	q	10	24	
Cyhalothrin	C_23_H_19_ClF_3_NO_3_	449.9	197.0	141.0	Q	10	24	12.01, 12.13
			197.0	161.0	q	5	24	
Permethrin	C_21_H_20_Cl_2_O_3_	391.3	183.1	168.1	Q	15	18	12.66, 12.74
			183.1	153.0	q	10	18	
Cyfluthrin	C_22_H_18_Cl_2_FNO_3_	434.3	226.0	206.0	Q	18	15	13.05, 13.11, 13.18
			198.9	170.1	q	18	25	
Cypermethrin	C_22_H_19_Cl_2_NO_3_	416.3	163.0	91.0	Q	18	10	13.29, 13.36, 13.44
			163.0	127.0	q	18	5	
Flucythrinate	C_26_H_23_F_2_NO_4_	451.5	156.9	107.1	Q	18	15	13.39, 13.53
			198.9	157.0	q	18	15	
Tau-fluvalinate	C_26_H_22_ClF_3_N_2_O_3_	502.9	250.0	55.0	Q	37	40	14.19, 14.24
			181.0	152.0	q	37	40	
Fenvalerate	C_25_H_22_ClNO_3_	419.9	167.0	125.1	Q	37	5	14.02, 14.20
			224.9	119.0	q	37	15	
Deltamethrin	C_22_H_19_Br_2_NO_3_	505.2	252.9	93.0	Q	37	15	14.73
			250.7	172.0	q	37	5	

^a^ Q is quantification ion transition and q is confirmation ion transition.

### Optimization of extraction solvents and clean up sorbents

**Figure 1:**
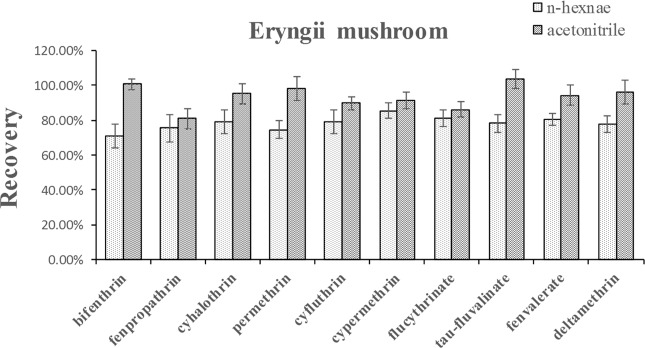
Effect of acetonitrile and *n*-hexane as extraction solvents for the target compounds in eryngii mushroom at 100 μg kg^−1^ level (n = 3).

The choice of suitable solvent and sorbent has huge influence on the recoveries. Therefore, the solvents and sorbents need to be optimized. Firstly, the extraction solvent was studied. Acetonitrile was frequently used for pesticide multi-residue analysis with advantages including, less co-extracted matrix components, higher recoveries, etc.^2,24,27,33^. Meanwhile, pyrethroid insecticides have high solubility in *n*-hexane at 20 °C. Therefore, the recoveries of acetonitrile and *n*-hexane as extraction solvents were compared. As shown in Fig. 1, taking eryngii mushroom as an example, the recoveries of the ten pyrethroid insecticides at a spiked level of 100 μg kg^−1^ using acetonitrile as the extraction solvent was significantly higher than that of *n*-hexane. Consequently, acetonitrile was selected as the extraction solvents in further study.

**Figure 2:**
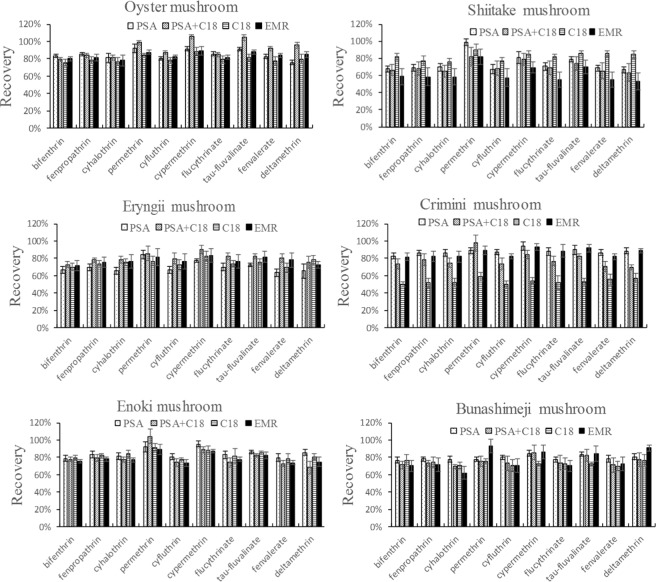
Effects of different sorbents for the targeted compounds in different matrix at 100 μg kg^−1^ level (n = 5).

To achieve a satisfactory effect, we evaluated the different types of sorbents at a spiked level of 100 μg kg^−1^. Test A was carried out using 50 mg PSA + 150 mg MgSO_4_, test B using 20 mg PSA + 30 mg C18 + 150 mg MgSO_4_, test C using 50 mg C18 + 150 mg MgSO_4_, and test D using an Enhanced Matrix Removal-Lipid (EMR-Lipid). Meanwhile, the four matrix standards were prepared from each dispersive solid phase extraction (dSPE) cleanup technique so that each (C18, PSA or PSA + C18, etc.) could be tested against standards with the same composition of matrix compounds. For the dSPE, PSA is mainly applied to adsorb various polar matrix components from non-polar samples like organic acids and pigments. Conversely, C18 is mainly used to remove non-polar and medium-polar compounds from the polar samples^2,33,34^. Particularly, the dSPE EMR is applied to remove the lipid^35^. As shown in Fig. 2, the recovery and RSD were both satisfied when the four different types of sorbents were used in the oyster mushroom. Nevertheless, when C as sorbent was used in shiitake mushroom, the recoveries of ten target compounds was satisfactory. Meanwhile, A as sorbent was used in bunashimeji mushroom, the recoveries was satisfactory. For the crimini mushroom and enoki mushroom, the recovery and RSD were both satisfied when sorbent A, sorbent B and sorbent D were used. PSA is relatively expensive than C18. Therefore, considering the efficacy and cost of each sorbent, 20 mg PSA + 30 mg C18 + 150 mg MgSO_4_ was ultimately selected as sorbent for oyster mushroom and eryngii mushroom extracts. 50 mg PSA + 150 mg MgSO_4_ was used as the sorbent for crimini mushroom, enoki mushroom and bunashimeji mushroom extracts, while for the shiitake mushroom requires used 50 mg C18 + 150 mg MgSO_4_ purification.

### Matrix effects

The ionization of some pesticides may be significantly affected by the presence of substances, which are derived from samples^36^. In 1993, matrix effect was first explained by Erney and co-workers, and their study suggested that the response of one organic base decreased as the concentration of other bases increased^37^. The matrix effects can greatly affect the reproducibility and accuracy of the method. Thus, matrix effect was studied in edible mushrooms. Generally speaking, the matrix effect was ignored if the value was between −10% and 10%; the matrix effect was defined as suppression if the value was lower than −10%; the matrix effect was defined as enhancement if the value was higher than 10%^23,24^. As shown in Table 2, the matrix effects obviously enhance the response of the instrument in all matrices. And the slope ratios of matrix/*n*-hexane were in the range of 1.47–2.77. In order to eliminate the matrix effect and determine more accurate results for each target compound concentration in all samples, the matrix-matched calibration curves were selected to calibrate the GC-MS/MS system.

**Table 2:** Comparison of matrix-matched calibration and solvent calibration at 10–1000 μg kg^−1^.

Compound	Matrix	Regression equation	R^2^	Matrix effect (%)	LOD (μg kg^−1^)	LOQ (μg kg^−1^)
Bifenthrin	n-Hexane	y = 917.43×−1669.74	0.9992	—	—	—
	Oyster mushroom	y = 1378.65×+11014.79	0.9998	50.27	0.037	0.122
	Shiitake mushroom	y = 1406.06×+4712.20	0.9995	53.26	0.071	0.237
	Eryngii mushroom	y = 1410.36×−963.07	0.9992	53.73	0.021	0.069
	Crimini mushroom	y = 1350.62×+6803.68	0.9994	47.22	0.016	0.054
	Enoki mushroom	y = 1366.83 + 17846.90	0.9989	48.98	0.038	0.126
	Bunashimeji mushroom	y = 1419.98 + 5624.00	0.9999	54.78	0.025	0.083
Fenpropathrin	n-Hexane	y = 69.32×−1602.22	0.9976	—	—	—
	Oyster mushroom	y = 112.28×+156.06	0.9999	61.97	0.148	0.492
	Shiitake mushroom	y = 114.69×−108.54	0.9994	65.45	0.161	0.537
	Eryngii mushroom	y = 113.88×−558.57	0.9991	64.28	0.034	0.112
	Crimini mushroom	y = 107.86×+21.11	0.9995	55.60	0.105	0.351
	Enoki mushroom	y = 107.80×+871.59	0.9993	55.51	0.116	0.385
	Bunashimeji mushroom	y = 111.61×−255.32	0.9999	61.01	0.754	2.515
Cyhalothrin	n-Hexane	y = 94.09×−2757.26	0.9952	—	—	—
	Oyster mushroom	y = 185.33×−1579.27	0.9998	96.97	1.211	4.036
	Shiitake mushroom	y = 203.46×−1374.18	0.9990	116.24	0.304	1.014
	Eryngii mushroom	y = 195.85×−2845.42	0.9982	108.15	0.812	2.705
	Crimini mushroom	y = 178.54×−1265.32	0.9997	89.75	0.138	0.458
	Enoki mushroom	y = 181.44×−661.70	1.0000	92.84	0.230	0.767
	Bunashimeji mushroom	y = 176.70×−1785.85	0.9996	87.80	0.237	0.791
Permethrin	n-Hexane	y = 229.92×−1715.55	0.9990	—	—	—
	Oyster mushroom	y = 380.94×+9262.22	0.9992	65.68	0.059	0.196
	Shiitake mushroom	y = 398.28×+11871.62	0.9984	73.23	0.032	0.108
	Eryngii mushroom	y = 382.27×+2916.55	0.9993	66.26	0.026	0.086
	Crimini mushroom	y = 366.04×+8864.70	0.9991	59.20	0.015	0.051
	Enoki mushroom	y = 367.54×+8021.24	0.9989	59.86	0.036	0.119
	Bunashimeji mushroom	y = 384.23×+7303.04	0.9995	67.11	0.025	0.083
Cyfluthrin	n-Hexane	y = 93.91×−2395.75	0.9985	—	—	—
	Oyster mushroom	y = 234.30×−3149.71	0.9995	149.49	0.488	1.625
	Shiitake mushroom	y = 249.29×−2914.76	0.9987	165.46	1.163	3.876
	Eryngii mushroom	y = 230.07×−4354.54	0.9976	144.99	0.127	0.424
	Crimini mushroom	y = 212.24×−3468.49	0.9989	126.00	0.103	0.344
	Enoki mushroom	y = 205.24×−2701.42	0.9994	118.55	0.363	1.211
	Bunashimeji mushroom	y = 214.95×−3724.08	0.9983	128.89	1.073	3.576
Cypermethrin	n-Hexane	y = 204.64×−7437.40	0.9936	—	—	—
	Oyster mushroom	y = 475.19×+1202.65	0.9998	132.21	0.065	0.218
	Shiitake mushroom	y = 506.74×+13.76	0.9989	147.63	0.059	0.195
	Eryngii mushroom	y = 475.26×−1499.63	0.9983	132.24	0.126	0.419
	Crimini mushroom	y = 443.91×+2479.19	0.9993	116.92	1.259	4.198
	Enoki mushroom	y = 430.10×+1144.36	0.9998	110.17	0.723	2.411
	Bunashimeji mushroom	y = 451.72×+740.57	0.9989	120.74	1.221	4.068
Flucythrinate	n-Hexane	y = 388.40×−12880.95	0.9945	—	—	—
	Oyster mushroom	y = 1030.38×−7947.70	0.9999	165.29	0.451	1.503
	Shiitake mushroom	y = 1077.43×−8247.59	0.9991	177.40	0.222	0.739
	Eryngii mushroom	y = 1020.13×−9961.48	0.9988	162.65	0.869	2.896
	Crimini mushroom	y = 954.05×−8132.86	0.9995	145.64	0.070	0.233
	Enoki mushroom	y = 940.39×−3366.13	0.9999	142.12	0.149	0.496
	Bunashimeji mushroom	y = 970.90×−10710.4	0.9994	149.97	0.122	0.407
Tau-fluvalinate	n-Hexane	y = 23.03×−268.86	0.9987	—	—	—
	Oyster mushroom	y = 48.05×−1026.10	0.9964	108.64	0.468	1.560
	Shiitake mushroom	y = 54.90×−847.89	0.9961	138.38	0.355	1.183
	Eryngii mushroom	y = 49.10×−1231.91	0.9955	113.20	1.184	3.947
	Crimini mushroom	y = 43.50×−683.67	0.9969	88.88	0.604	2.015
	Enoki mushroom	y = 40.51×−732.66	0.9975	75.90	0.787	2.622
	Bunashimeji mushroom	y = 42.48×−867.74	0.9951	84.46	0.673	2.242
Fenvalerate	n-Hexane	y = 116.97×−5266.69	0.9906	—	—	—
	Oyster mushroom	y = 298.44×−5894.21	0.9991	155.14	0.669	2.229
	Shiitake mushroom	y = 319.06×−5407.40	0.9984	172.77	1.669	5.565
	Eryngii mushroom	y = 298.29×−6626.48	0.9975	155.01	1.374	4.580
	Crimini mushroom	y = 277.95×−5612.92	0.9987	137.63	1.048	3.492
	Enoki mushroom	y = 270.36×−4366.27	0.9995	131.14	0.750	2.499
	Bunashimeji mushroom	y = 277.19×−5915.89	0.9984	136.98	0.219	0.730
Deltamethrin	n-Hexane	y = 31.82×−984.06	0.9953	—	—	—
	Oyster mushroom	y = 86.78×−2872.02	0.9958	172.72	1.210	4.032
	Shiitake mushroom	y = 78.89×−2031.72	0.9967	147.93	0.071	0.238
	Eryngii mushroom	y = 85.89×−1656.28	0.9976	169.92	1.312	4.373
	Crimini mushroom	y = 75.92×−534.92	0.9962	138.59	0.035	0.118
	Enoki mushroom	y = 72.69×−1603.40	0.9953	128.44	0.076	0.252
	Bunashimeji mushroom	y = 70.21×−992.51	0.9989	120.65	0.093	0.310

Matrix effect (%) = ((slope matrix/slope solvent) − 1) ×100.

### Linearity, LOD, and LOQ

The calibration curves in different edible mushrooms matrices were shown in Table 2. The linearity for each target compound in each edible mushroom matrix was satisfactory (*R*^2^ ≥ 0.9901 in all cases). The LODs of ten pyrethroid insecticides ranged from 0.015 to 1.67 μg kg^−1^, and LOQs ranged from 0.051 to 5.57 μg kg^−1^ in original samples. These values were similar to the values of other pesitcides reported in the literature^31,38,39^. And the LOQs for ten target compounds were much lower than the maximum residue limit (MRLs) (100–1000 μg kg^−1^) recommended by the EU, Japan, USA and China.

### Precision and accuracy

A recovery assay was performed to validate the performance of the proposed method. The blank samples of different matrices were spiked at three different concentrations (10, 100 and 1000 μg kg^−1^) and then determining them in quintuplicate. The method’s precision was expressed as the RSD. As indicated in Table 3, mean recoveries of ten target compounds were in the acceptable ranges of 80.8–97.7% with RSD_r_ of 1.0–8.4%, 81.5–103.6% with RSD_r_ of 1.8–8.4%, 72.8–97.5% with RSD_r_ of 1.4–7.0%, 81.4–102.2% with RSD_r_ of 2.4–8.9%, 75.6–100.0% with RSD_r_ of 2.4–9.0%, 75.0–103.3% with RSD_r_ of 1.0–8.4% for oyster mushroom, shiitake mushroom, eryngii mushroom, crimini mushroom, enoki mushroom and bunashimeji mushroom, respectively. In general, the mean recoveries of ten target compounds were 72.8–103.6% in all matrices, and the RSD_r_ (n = 5) and RSD_R_ (n = 15) values ranged from 1.0% to 9.0% and 3.1% to 13.0%, respectively. For the statistical analysis, one-way analysis of variance (ANOVA) at 95% confidence limits was used to compare the interday and intraday assay recoveries, and there were no significant differences between the interday and intraday assays. Therefore, the results indicated that the extract method and GC-MS/MS analysis can obtain a satisfactory precision and accuracy for residue analysis of these ten pyrethroid insecticides in edible mushrooms.

**Table 3:** Recoveries (n = 15, %), RSDr^a^ and RSD_R_^b^ (%) for target compounds from different matrices at three spiked levels.

		Oyster mushroom			Shiitake mushroom			Eryngii mushroom			Crimini mushroom			Enoki mushroom			Bunashimeji mushroom		
		0.01	0.1	1	0.01	0.1	1	0.01	0.1	1	0.01	0.1	1	0.01	0.1	1	0.01	0.1	1
Bifenthrin	Recovery	89.6	86.9	92.1	87.3	83.2	86.0	75.9	74.8	78.6	97.1	85.7	81.4	87.5	75.6	81.9	88.6	75.7	80.6
	RSD_r_^a^	7.3	3.3	5.5	8.1	5.8	2.7	3.7	3.1	3.4	2.4	4.6	7.9	3.6	5.0	4.9	1.7	3.9	3.6
	RSD_R_^b^	7.5	5.5	8.5	6.6	4.5	5.9	4.3	4.1	3.6	6.0	8.4	8.4	6.9	7.4	6.8	3.7	7.3	5.4
Fenpropathrin	Recovery	89.0	91.9	87.6	88.8	84.2	84.9	76.8	74.9	87.0	102.0	86.1	84.9	79.7	78.7	83.7	91.8	75.8	82.7
	RSD_r_^a^	6.6	3.0	3.4	4.2	5.4	2.8	3.5	2.9	3.8	2.5	7.5	8.9	7.6	6.6	5.7	1.8	2.2	4.4
	RSD_R_^b^	10.5	6.1	4.7	4.3	6.7	5.4	3.4	4.1	3.5	8.1	9.1	6.3	7.1	6.1	7.2	3.6	4.4	13.0
Cyhalothrin	Recovery	97.7	94.5	90.1	89.6	86.0	87.6	74.9	72.8	88.5	96.3	84.7	90.7	91.7	74.8	87.6	97.9	78.5	84.8
	RSD_r_^a^	1.0	5.0	8.4	5.0	3.4	2.5	3.8	2.6	2.0	4.9	4.2	7.4	5.0	4.3	5.5	5.5	2.9	5.9
	RSD_R_^b^	4.7	7.9	7.5	3.8	7.1	5.3	4.2	3.1	6.4	5.3	7.0	8.1	6.1	5.6	6.5	7.1	5.6	5.5
Permethrin	Recovery	91.9	84.4	80.8	91.0	85.3	83.2	97.2	80.8	87.7	89.7	85.9	83.5	92.6	78.5	84.4	98.8	82.1	87.2
	RSD_r_^a^	4.2	3.2	8.0	3.8	5.7	4.1	3.8	4.6	4.7	3.6	3.0	6.9	5.2	6.8	5.6	2.8	3.6	4.1
	RSD_R_^b^	6.3	5.7	7.3	4.7	5.7	6.2	5.0	5.2	5.4	8.2	6.3	7.1	7.2	5.6	7.5	5.6	7.3	5.8
Cyfluthrin	Recovery	96.9	95.6	90.1	87.0	83.5	88.7	74.0	78.4	77.8	97.6	82.6	83.1	86.2	81.0	86.6	94.5	82.2	83.9
	RSD_r_^a^	3.6	4.1	7.9	4.7	5.0	3.7	2.3	1.9	4.5	5.0	5.5	7.8	9.0	6.7	6.9	4.1	3.0	8.0
	RSD_R_^b^	4.4	4.3	6.2	7.6	5.7	6.2	3.3	4.0	7.3	6.4	9.2	8.5	8.1	7.4	6.7	5.0	6.3	9.4
Cypermethrin	Recovery	95.6	95.5	89.0	103.6	92.5	90.7	97.5	75.8	80.8	93.5	88.5	88.3	100.0	89.1	83.9	103.3	80.0	80.8
	RSD_r_^a^	5.9	5.5	8.4	1.8	8.7	6.0	1.4	4.3	4.6	4.0	4.5	8.3	2.5	3.4	6.0	2.8	2.4	6.4
	RSD_R_^b^	4.7	6.0	7.6	4.3	6.0	6.8	6.0	7.1	6.5	8.3	6.7	6.3	5.3	5.0	3.5	3.5	5.9	9.0
Flucythrinate	Recovery	90.3	84.9	86.1	85.2	81.5	88.6	77.6	76.1	85.4	95.6	88.3	85.6	82.3	83.6	86.3	94.4	77.7	85.9
	RSD_r_^a^	2.6	5.8	4.3	3.9	5.4	3.8	6.3	3.8	4.2	2.4	4.3	5.9	5.6	2.4	5.9	5.2	4.6	6.2
	RSD_R_^b^	3.4	10.5	8.6	5.8	7.6	4.4	7.3	5.9	8.7	6.7	5.0	7.0	7.4	4.1	5.8	7.2	6.6	9.3
Tau-fluvalinate	Recovery	94.4	94.0	92.4	93.8	87.9	101.2	79.9	83.9	79.3	102.2	82.8	90.2	89.9	81.9	97.8	93.6	77.3	83.9
	RSD_r_^a^	3.6	5.8	4.9	4.9	4.4	2.8	4.8	4.1	5.7	2.6	5.1	7.0	8.1	6.0	6.8	3.4	4.1	7.0
	RSD_R_^b^	4.7	6.5	4.3	5.4	6.9	5.0	9.1	10.4	3.8	4.5	5.6	7.0	5.8	7.4	7.2	7.7	4.9	10.2
Fenvalerate	Recovery	92.9	88.4	87.7	90.5	84.5	103.6	75.9	81.9	91.7	98.3	82.9	101.4	79.0	82.4	94.7	90.4	81.4	91.4
	RSD_r_^a^	2.3	4.5	4.7	8.3	3.7	2.9	4.7	5.0	5.4	5.1	5.5	6.0	6.5	6.8	4.0	4.8	6.1	2.5
	RSD_R_^b^	5.7	7.5	6.7	7.2	5.2	5.7	5.5	8.5	5.7	5.2	5.4	5.9	6.7	6.1	8.0	6.2	7.2	6.0
Deltamethrin	Recovery	91.3	88.4	77.6	97.9	85.8	92.4	91.6	81.2	76.8	100.5	89.2	83.6	96.5	91.6	81.8	91.3	75.0	83.6
	RSD_r_^a^	6.5	7.7	4.9	4.4	5.4	3.1	7.0	6.9	7.5	3.5	3.2	3.7	2.4	5.4	7.1	2.5	2.3	4.8
	RSD_R_^b^	4.6	7.6	6.0	4.8	6.6	5.5	4.6	7.0	8.1	4.2	4.8	7.1	5.9	8.5	12.4	8.6	7.5	8.2

The recovery is the mean recovery.

^a^Intra-day (n = 5).

^b^Inter-day (n = 15).

### Application to real samples

The proposed method was applied to monitor trace levels of each target compounds in real samples to demonstrate the effectiveness and applicability. These samples were purchased from markets in Anhui Province (China). A total of 90 samples (20 oyster mushroom samples, 20 shiitake mushroom samples, 10 eryngii mushroom samples, 20 crimini mushroom samples, 10 enoki mushroom samples, and 10 bunashimeji mushroom samples) were analyzed. As shown in Table 4, only two positive oyster mushroom samples and three positive crimini mushroom samples were detected containing cypermethrin in the range of 11–43 μg kg^−1^. However, the presence of cypermethrin doesn’t pose a threat to the consumer, because they are below the MRLs settled by EU (50 μg kg^−1^ for oyster mushroom and crimini mushroom), China (500 μg kg^−1^ for oyster mushroom and crimini mushroom) and Japan (50 μg kg^−1^ for crimini mushroom and 500 μg kg^−1^ for oyster mushroom). But the residual concentration of cypermethrin in some crimini mushroom samples is very close to MRL settled by EU and Japan. Therefore, detection of cypermethrin residues in mushrooms should be strengthened. However, ten pyrethroid insecticides were not found in most of tested samples.

**Table 4:** Concentration levels of ten pyrethroid insecticides in edible mushroom samples from market in Anhui Province.

Samples	Number of samples	Positive sample ratio^a^	Concentration (μg kg^−1^)									
			Bifenthrin	Fenpropathrin	Cyhalothrin	Permethrin	Cyfluthrin	Cypermethrin	Flucythrinate	Tau-fluvalinate	Fenvalerate	Deltamethrin
Oyster mushroom	20	2(10%)	<LOQ	<LOQ	<LOQ	<LOQ	<LOQ	24/43^b^	<LOQ	<LOQ	<LOQ	<LOQ
Shiitake mushroom	20	0(0)	<LOQ	<LOQ	<LOQ	<LOQ	<LOQ	<LOQ	<LOQ	<LOQ	<LOQ	<LOQ
Eryngii mushroom	10	0(0)	<LOQ	<LOQ	<LOQ	<LOQ	<LOQ	<LOQ	<LOQ	<LOQ	<LOQ	<LOQ
Crimini mushroom	20	3(15%)	<LOQ	<LOQ	<LOQ	<LOQ	<LOQ	11/16/35^b^	<LOQ	<LOQ	<LOQ	<LOQ
Enoki mushroom	10	0(0)	<LOQ	<LOQ	<LOQ	<LOQ	<LOQ	<LOQ	<LOQ	<LOQ	<LOQ	<LOQ
Bunashimeji mushroom	10	0(0)	<LOQ	<LOQ	<LOQ	<LOQ	<LOQ	<LOQ	<LOQ	<LOQ	<LOQ	<LOQ

^a^Number of positive sample (positive sample ratio).

^b^The result of positive samples.

In conclusion, in the present study, a simple, reliable and highly sensitive residue analytical method for the simultaneous determination of ten pyrethroid insecticides in six edible mushrooms using GC-MS/MS was developed. The results showed satisfactory validation parameters in the field of linearity, lower limits, accuracy, and precision. The LOQs were below MRLs recommended by EU, China and Japan in all mushroom matrices. The method has strong matrix effect, but it was successfully normalized using matrix-matched calibration. Therefore, this method may be a useful technique for monitoring pyrethroid insecticide residues in edible mushroom samples.

## Materials and Methods

### Reagents and chemicals

Insecticide analytical standards were supplied from the National Institute of Metrology (Beijing, China) and were of more than 98% purity. Chromatography grade acetonitrile and *n*-hexane were achieved from Honeywell International Inc. (New Jersey, USA). The anhydrous magnesium sulfate (MgSO_4_) and sodium chloride (NaCl) were bought from Beijing Chemical and Reagent Company (Beijing, China). The sorbents of primary secondary amine (PSA) and octadecylsilane (C_18_) were bought from Agela Technologies Inc. (Beijing, China), and Agilent Bond Elut dSPE EMR-Lipid was also bought from Agela Technologies Inc.

Stocks solutions (1000 mg L^−1^) of each insecticide standard were prepared in *n*-hexane. A mixed stock standard solution of 100 mg L^−1^ containing bifenthrin, fenpropathrin, cyhalothrin, permethrin, cyfluthrin, cypermethrin, flucythrinate, tau-fluvalinate, fenvalerate, and deltamethyrin was prepared by mixing ten stock solutions in equal volume. Subsequently, several standard solutions (10, 50, 100, 200, 500, and 1000 μg L^−1^) were prepared from the mixed stock solution by serial dilution with *n*-hexane. The matrix-matched standard solutions (10, 50, 100, 200, 500, and 1000 μg L^−1^) were similarly prepared by adding the blank sample extracts (oyster mushroom, shiitake mushroom, eryngii mushroom, crimini mushroom, enoki mushroom and bunashimeji mushroom) to each serially diluted standard solution. For the preparation of matrix-matched standard, the method was that appropriate volumes of work standard solution was firstly dried under nitrogen and then redissolved by 1 mL blank sample extract. All solutions were stored at −20 °C in the dark.

### Instruments and chromatographic conditions

All sample analyses used an Agilent intuvo 9000 gas chromatograph coupled with a 7000D triple quadrupole mass spectrometer. Separations were performed using Agilent Technologies Capillary Column HP-5MS phenylmethyl siloxane fused-silica capillary analytical column (30 m length × 0.25 mm i.d. × 0.25 μm film thickness). A helium (purity 99.99%) was employed as carrier gas and the flow rate was 1.0 mL min^−1^. The temperature of the injection port was 280 °C. The column temperature was initially at 70 °C for 1 min, increased to 120 °C at the rate of 40 °C min^−1^, and increased to 200 °C at the rate of 30 °C min^−1^, then increased to 240 °C at the rate of 10 °C min^−1^, and then increased to 300 °C at the rate of 20 °C min^−1^, and holding for 3.7 min. A volume of 1 μL was injected in the splitless mode.

The mass spectrometer was performed in electron ionization mode with an ionizing energy of 70 eV. The electron multiplier voltage was 1300 V. The transfer line, manifold and ionization source temperatures were 280, 40 and 250 °C, respectively. A solvent delay was 8 min. The mass spectrometer mode was set at multiple reaction monitoring (MRM) to collect data. The concrete MS/MS parameters for all the analytes listed in Table 1.

### Sample preparation

**Figure 3:**
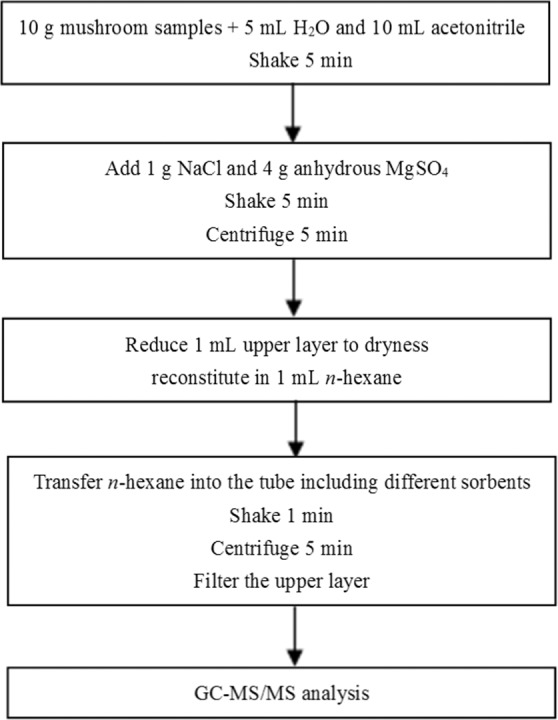
Workflow of sample preparation.

Figure 3 shows the workflow of the sample preparation procedure. For the cleanup procedure, the 20 mg PSA and 30 mg C18 were selected to clean up the oyster mushroom and eryngii mushroom; the 50 mg PSA was used to purify the crimini mushroom, enoki mushroom and bunashimeji mushroom, and 50 mg C18 was used to clean up the shiitake mushroom.

For the Agilent Bond Elut EMR-Lipid clean up, the extract procedure is the same as above. 5 mL Milli-Q water was added into EMR-Lipid tube to activate the sorbent. Then, 5 mL upper layer (acetonitrile) was added into the tube. The tube was vortexed for 5 min and then centrifuged for 5 min at relative centrifugal force (RCF) 3913 × *g*. Subsequently, 5 mL upper layer was transferred into the EMR-Polish tube that containing 2 g salt (1:4 NaCl: MgSO_4_). The tube was vortexed for 5 min and centrifuged for 3 min at RCF 2811 × *g*. Then, 1 mL upper layer (acetonitrile) was reduced to dryness under a gentle stream of nitrogen at room temperature. The residue was reconstituted in 1 mL *n*-hexane and was filtered with 0.22-μm filters for GC-MS/MS analysis.

### Method validation

The developed method was validated by fortifying blank mushroom samples at three different levels (10, 100, and 1000 μg kg^−1^). Recovery assays were performed to determine the accuracy and precision of the method. For determination of the accuracy, five replicates of each fortification level were prepared on three different days (n = 15 in total). However, the intra-day and inter-day relative standard deviation (RSD) were also investigated as the method precision. The linear regression equations of each target compound were achieved from the peak area ratios plotted against its respective concentrations (10–1000 μg kg^−1^). The linearity was presented as correlation coefficient (*R*^2^). The limit of detection (LOD) for each target compound was defined as the minimum spiking level that produced a chromatogram peak with signal-to-noise (peak to peak) ratio of 3, and the limit of quantification (LOQ) was defined as a signal-to-noise ratio of 10^23^.
